# 

**Published:** 1897-06

**Authors:** 


### CONTENTS.

ORIGINAL COMMUNICATIONS. PAGE

Root-Perforation: A New Method of Treatment. By JOHN GIRDWOOD, D.D.S., L.D.S., Edinburgh, Scotland . . . 357

Some Points in the Antiseptic Treatment of Root-Canals. By J. MORGAN HOWE, M.D., M.D.S. . . . 364

Treatment of Devitalized Teeth. By LOUIS JACK, D.D.S. . . . 370

To what Extent is Typhoid Fever Preventable? By J. L. HILDRETH, M.D., Cambridge, Mass. . . . 373

Electricity in Alveolar Inflammations. By WILLIAM ROLLINS . . . 387

Dr. Robinson *versus* Dr. Younger, in Reference to Implantation. By LOUIS JACK, D.D.S. . . . 388

New Field suggested by Dr. Williams's Paper. By DR. G. A. MILLS, New York . 390

ABSTRACTS AND TRANSLATIONS.

The Microscopical Aspect of Certain Lesions induced by Dental Caries. By A. HOPEWELL SMITH, L.R.C.P. (Lond.), M.R.C.S., L.D.S. (Eng.) . . . 392

REPORTS OF SOCIETY MEETINGS.

The New York Institute of Stomatology . . . 401

EDITORIAL.

Reply to Criticisms . . . 415

Dr. Farrar's Second Volume . . . 416

BIBLIOGRAPHY . . . 417

OBITUARY.

Frank Abbott, M.D. . . . 418

CURRENT NEWS.

Resolution of the Odontological Society of Pennsylvania . . . 420

Pennsylvania State Dental Examining Board . . . 420

Pennsylvania State Dental Society . . . 420

